# Exclusive breastfeeding practice during COVID-19 pandemic in West Java Indonesia: A cross-sectional study

**DOI:** 10.1371/journal.pone.0303386

**Published:** 2024-05-23

**Authors:** Apriningsih, Laily Hanifah, Nanang Nasrulloh

**Affiliations:** Faculty of Health Science, Universitas Pembangunan Nasional Veteran Jakarta, Depok, Indonesia; UiA: Universitetet i Agder, NORWAY

## Abstract

**Background:**

The achievement towards 100% exclusive breastfeeding still a challenge in many countries despite adverse impacts due to the absence of exclusive breastfeeding. One consequence from the low practice of exclusive breastfeeding is malnutrition, including stunting that can be prevented by providing optimal food to infants, starting with providing exclusive breastfeeding from birth to 6 months of age. However, the practice of exclusive breastfeeding still low and it is suspected that this practice also decreased during the COVID- 19 pandemic. This study aims to analyze the determinants of exclusive breastfeeding in sub-urban areas during the COVID-19 pandemic.

**Methods:**

This study using cross sectional design conducted from interviewing 206 mothers in 2022 who meet the inclusion criteria, consisted of breastfeeding their babies in the last 1 year and live in Sub-urban area in Depok City, West Java. Multiple binary logistic regression used to measure the association and strength between independent variables with the outcome variable. Independent variables with a p-value < 0.25 during the Chi-square test were included in the logistic regression model.

**Results:**

Prevalence of exclusive breastfeeding and early initiation of breastfeeding (EIB) was 58.3% and 57.8% respectively. Factors associated with exclusive breastfeeding practices are education, employment status, knowledge and attitude about exclusive breastfeeding, self-efficacy in providing exclusive breastfeeding, EIB practice, and eating pattern. From multivariate analysis, it was found that the dominant factors to exclusive breastfeeding are EIB.

**Conclusions:**

The study highlights the importance of improving exclusive breastfeeding practice through early initiation of breastfeeding, mother’s knowledge, education and self-efficacy. Therefore, health promotion and education should emphasize the importance of those factors, supported by the health policy and massive campaign as a key success in exclusive breastfeeding.

## Introduction

The achievement towards 100% exclusive breastfeeding still a challenge in many countries, even though there are many adverse impacts due to the absence of exclusive breastfeeding. One consequence from the low practice of exclusive breastfeeding is malnutrition, including stunting. The prevalence of child stunting worldwide indicated by a poor height-for-age ratio, was 22.9% or 154.8 million children under the age of five in 2016 [1]. The World Health Organization (WHO) Child Growth Standards median, which indicates a restriction of a child’s potential growth, is used to quantify stunting [2].

Indonesia is one of 17 countries with triple burden of malnutrition, namely stunting (30.8%), wasting (12.1%) and obesity (11.9%) [3]. Indonesia is the country with the fifth largest number of stunted children in the world. Indonesian Family Life Survey data reported that the prevalence of stunting increased from 29.7% in 2000 to 32.6% in 2014 [4]. Although the latest survey found a decrease in stunting in Indonesia to 26.1% in 2016 and 24.4% in 2021, this figure still does not reach the targeted rate of 14% [5]. According to the UNICEF and WHO framework, the direct causes of stunting are suboptimal intake of nutritious food and infectious diseases [6,7].

One mean of stunting prevention is by ensuring adequate food to infants, by providing exclusive breastfeeding from birth to 6 months of age and complementary foods from 6 months of age onwards along with breastmilk until the age of 2 years [8]. Based on previous studies, there are differences in the results of the prevalence of exclusive breastfeeding practices among urban and rural areas. Previous studies found a higher rate of exclusive breastfeeding practices in urban areas [9,10]. On the contrary, other study found the rate of exclusive breastfeeding practices in urban areas is lower than in rural areas [11].

Area of residence, maternal ethnicity, maternal occupation, maternal smoking status, parity, gestational age, husbands’ support for breastfeeding, and rooming-in practice were factors associated with exclusive breastfeeding, according to research conducted in Malaysia [12]. Research found maternal age, parity, education, health insurance, mode of birth, inadequate breastfeeding support, lack of early breastfeeding initiation, birth attendant, perceived healthcare professionalism and attention, facility room cleanliness, the timing of birth, and place of birth were significantly associated with exclusive breastfeeding [13]. In the previous study, factors such as annual family income and mothers who received breastfeeding education were related to a higher prevalence of EBF [14].

The COVID-19 pandemic has hampered many health programs including the promotion and education of exclusive breastfeeding in health services, this is assumed affect the exclusive breastfeeding practices. COVID-19-positive mothers may experience limited professional support after birth to help them deal with problems such as difficult latching, sore nipples, thrust infections and tongue ties [15]. Some evidence suggests that several health facilities are improperly separating newborn from mothers and discouraging breastfeeding because of fears of COVID-19 transmission through breastmilk that might result in a decline in early initiation of breastfeeding and absent in providing colostrum as the child’s first natural vaccine and exclusive breastfeeding [16].

Therefore, this study was conducted to examine the factors associated with exclusive breastfeeding practices during the COVID-19 pandemic in one sub-urban area in Depok City, West Java. This study aims to analyze the factors associated with exclusive breastfeeding practices during the COVID-19 pandemic in sub-urban areas of Depok City, West Java. Depok City is one of the sub-urban areas located in a province with a high prevalence of stunting (31.1%), in West Java [3].

## Methods

### Ethical consideration

The protocol for this study was approved by the Universitas Pembangunan Nasional Veteran Jakarta Health Research Ethics Committee under the number 378/VIII/2022/KEPK. The date approval is on 24^th^ August 2022. Written informed consent was given to respondents before taking data.

### Study area

The study was held in Limo and Sawangan Districts of Depok City, West Java province, Indonesia by using cross-sectional method. Surveys of the cross-sectional type (where the information on cause and effect is simultaneously gathered, and the time sequence cannot be determined) are considered to be hypothesis-generating studies [17].

### Participants

The population of this study are mothers with infants aged 0 to 12 months in the Limo and Sawangan Districts of Depok City, West Java. Data collected from 26 August to 2 October 2022.

**Fig 1:**
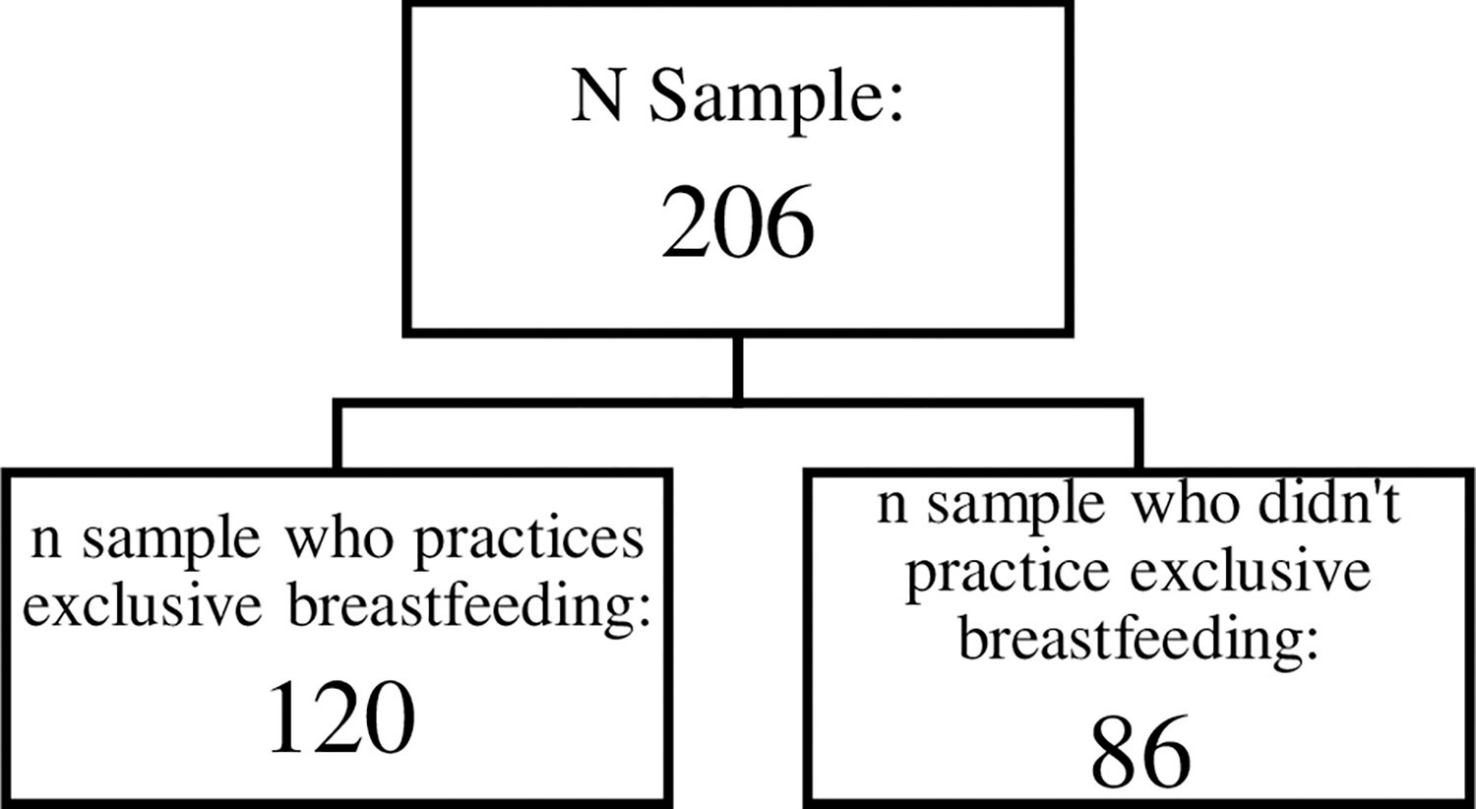
Sample flowchart.

The study sample is mothers who have had or are having babies aged 0–12 months in Limo and Sawangan sub-districts of Depok City, West Java, who meet the inclusion criteria, consisted of breastfeeding their babies in the last 1 year, residing in Limo and Sawangan sub-districts of Depok City, and are in good health. The sample size of 206 consisted of 120 who practice exclusive breastfeeding and 86 who did not practice exclusive breastfeeding as shown in Fig 1. The minimum sample size calculation was taken using the Lemeshow formula using P1 (0.043) and P2 (0.167) from previous research [18] for a two-proportion difference test as follows:

$$n=\frac{{\{Z}_{1-\alpha /2}\sqrt{2\bar{P}(1-\bar{P})}+Z_{1-\beta }\sqrt{P_{1}(1-P_{1})+P_{2}(1-P_{2})}\}}{{\left(P_{1}-P_{2}\right)}^{2}}$$

n = minimum sample size

Z1−a2 = Z score for level of confidence α = 5%

*Z*_1−*β*_ = Z score for power of the test β = 80%

P1 = Proportion of exclusive breastfeeding among high-risk group (0.043)

P2 = Proportion of exclusive breastfeeding among non-high-risk group (0.167)

### Data and instruments

There are some efforts taken to address potential bias. It started with following the ethical guidelines established by the institutional research committee in all procedures involving human beings. The research team used a standard questionnaire and informed consent form when they visit the participant’s house.

The variables examined include both dependent and independent factors. The dependent variable used is exclusive breastfeeding practice during the pandemic, measured by four questions: early breastfeeding initiation, breastfeeding until the age of six months, and not providing complementary foods/drinks/substitutes for breast milk, unless drugs or vitamins are prescribed by a doctor. Meanwhile, independent variables were the mother’s age was measured by asking the mother’s age in the current year, the level of education was classified into two levels, family income was classified into two categories based on the value of the minimum wage of Depok city in 2022 (Rp.4,377,231.93), the mother’s employment status was classified into two: employed or unemployed, maternal nutrition status was grouped based on WHO criteria on nutritional status using BMI then classified into two categories, poor and good (poor consisted of BMI under 18 Kg/m^2^ and above 25), good for BMI from 18.5 to 24.9 Kg/m^2^, location of birth (primary health center, hospital and others), Food Frequency Questionnaire score was divided into 2 groups: good (≥median score) and poor (<median score), knowledge, attitude, and self-efficacy was grouped by good and poor, whether infected by COVID-19 or not, whether consuming traditional foods that increase breast milk and doing breast care to enhance breast milk production.

The statistical analysis was performed using the statistical program SPSS version 25 to clean, recode and analyzed the collected data. Multiple binary logistic regression used to measure the association and strength between independent variables with the outcome variable. Independent variables with a p-value < 0.25 during the Chi-square test were included in the logistic regression model to assess the effect of confounder. A backward stepwise method was used in the selection process to build the logistic regression model. The strengths of association were explained with odd ratios (OR)

## Result

The characteristics of respondents in this study were mostly aged 20–35 years old (74.8%), primary and high school graduated (86.9%), income below or similar as the minimum wages in Depok City (58.7%). Most respondents were unemployed (54.4%), had a poor nutritional status during pregnant (70.4%) with a good eating pattern frequency (53.9%) and gave birth to their children in midwives clinic or home (39.8%) as shown in Table 1 below.

**Table 1:** Respondent’s characteristics.

Variables	N (n = 206)	Percent Prevalences
**Age**		
<20 & >35	52	25.2
20–35	154	74.8
**Education**		
Graduated from primary school and high school	179	86.9
Academy, bachelor’s degree and master degree	27	13.1
**Household Income**		
≤ RMW Depok City (≤ Rp.4,377,231.93)	121	58.7
> RMW Depok City (>Rp.4,377,231.93)	85	41.3
**Employment Status**		
Employed	94	45.6
Unemployed	112	54.4
**Nutrition Status During Pregnancy (BMI)**		
Poor	145	70.4
Good	61	29.6
**Eating Pattern Frequency Score using FFQ**		
Poor	95	46.1
Good	111	53.9
**Delivery Places**		
Others (midwife clinic or home)	88	42.7
Primary Health Center	36	17.5
Hospital	82	39.8

*RMW: Regional Minimum Wage.

Table 2 shows that more than half mothers have good knowledge and attitude on exclusive breastfeeding and early initiation of breastfeeding and good self-efficacy.

**Table 2:** Mother’s knowledge, attitude and self-efficacy related to exclusive breastfeeding practice and early initiation of breastfeeding (EIB).

Variables	N (n = 206)	Percent Prevalences
**Mother’s Knowledge Regarding Exclusive Breastfeeding and EIB Practice**		
Good	105	51.0
Poor	101	49.0
**Mother’s Attitude Regarding Exclusive Breastfeeding and EIB** **Practice**		
Good	113	54.9
Poor	93	45.1
**Mother’s Self-Efficacy**		
Good	178	86.4
Poor	28	13.6

Table 3 shows the prevalence of exclusive breastfeeding was 58.3%, while the Early Initiation of Breastfeeding (EIB) practices prevalence was 57.8%. The majority of respondents did not perform breast care to facilitate breast milk (78.2%), did not consume traditional herbs specifically to facilitate breast milk (74.3%). Around 8.7% mothers admitted to having COVID 19 during breastfeeding.

**Table 3:** Exclusive breastfeeding and EIB practice.

Variables	N (n = 206)	Percent Prevalences
**Practicing Exclusive Breastfeeding for 6 Months**		
Yes	120	58.3
No	86	41.7
**Practicing Early Initiation of Breastfeeding**		
Yes	119	57.8
No	87	42.2
**Performing Breast Care to Facilitate Breast Milk**		
Yes	45	21.8
No	161	78.2
**Consuming Special Traditional Herbs to Increase Breast Milk**		
Yes	53	25.7
No	153	74.3
**Have Been Exposed to COVID 19 while Breastfeeding**		
Yes	18	8.7
No	188	91.3

Table 4 shows that the results of the bivariate test using chi-square showed that the variables associated with exclusive breastfeeding practices among breastfeeding mothers were education, employment status, knowledge and attitude about exclusive breastfeeding, self-efficacy in providing exclusive breastfeeding, early initiative of breastfeeding practice, birth delivery place and eating pattern.

**Table 4:** Association of mother’s characteristics, knowledge, attitudes and self-efficacy with exclusive breastfeeding practices.

Variables	Exclusive Breastfeeding Practices				Bivariate Analysis		
	No		Yes		COR	95% CI	P-value
	n	%	n	%			
Age							
<20 & >35	23	44.2	29	55.8	1.15	0.61–2.2	0.797
20–35	63	40.9	91	59.1	1		
**Education**							
Graduated from primary school and high school	80	44.7	99	55.3	2.83	1.09–7.34	0.046**
Academy, bachelor’s degree and master degree	6	22.6	21	77.8	1		
**Employment Status**							
Employed	60	63.8	34	36.2	5.84	3.18–10.72	0.000**
Unemployed	26	23.2	86	76.8	1		
**Household Income**							
≤ RMW Depok City*	51	42.1	70	57.9	1.04	0.59–1.83	1.000
> RMW Depok City	35	41.2	50	58.8	1		
**Mother’s Knowledge**							
Poor	78	77.2	23	22.8	41.12	17.44–96.97	0.000**
Good	8	7.6	97	92.4	1		
**Mother’s Attitude**							
Poor	49	52.7	44	47.3	2.29	1.29–4.03	0.006**
Good	37	32.7	76	67.3	1		
**Mother’s Seld-efficacy**							
Poor	20	71.4	8	28.6	4.24	1.77–10.17	0.001**
Good	66	37.1	112	62.9	1		
**Practicing Early Initiation of Breastfeeding**							
No	74	85.1	13	14.9	50.76	21.94–117.4	0.000**
Yes	12	10.1	107	89.9	1		
**Nutrition Status During Pregnancy (BMI)**							
Poor	62	42.8	83	57.2	1.15	0.63–2.2	0.765
Good	24	39.3	37	60.7	1		
**Have Been Exposed to COVID 19 while Breastfeeding**							
Yes	11	61.1	7	38.9	2.37	0.88–6.38	0.135**
No	75	38.9	113	60.1	1		
**Consuming Special Traditional Herbs to Increase Breast Milk**							
No	61	39.9	92	60.1	0.74	0.39–1.39	0.443
Yes	25	47.2	28	52.8	1		
**Performing Breast Care to Facilitate Breast Milk Production**							
No	64	29.8	97	60.2	0.69	0.36–1.34	0.353
Yes	22	48.9	23	51.1	1		
**Eating Pattern**							
Poor	48	50.5	47	49.5	1.96	1.12–3.44	0.026**
Good	38	34.2	73	65.8	1		
**Delivery Places**							
Others (Home)	39	44.3	49	55.7	1.62	0.87–3.03	0.058**
Primary Health Center	20	55.6	16	44.4	2.55	1.14–5.68	
Hospital	27	32.9	55	67.2	1		

*RMW Depok City 2022: Rp.4,377,231.93.

**p<0.25 significance.

Table 4 also shows that mothers with lower education level were 2.83 times more likely did not perform exclusive breastfeeding compared to mothers with higher education. Mothers who work were 5.84 times more likely not practicing exclusive breastfeeding compared to those who did not work. Mothers with poor knowledge regarding exclusive breastfeeding and EIB practice were 41.12 more likely not doing exclusive breastfeeding than mothers with good knowledge. Mothers with poor attitudes and self-efficacy respectively were 2.29 and 4.24 times more likely not practicing exclusive breastfeeding, compared to mothers with good attitudes and self-efficacy. Mothers who did not performed early initiation of breastfeeding were 50.76 times more likely not practicing exclusive breastfeeding compared to those who performed EIB. Mothers who had a poor eating pattern were 1.96 more likely not practicing exclusive breastfeeding compared to those who had a good one. Mothers who give birth at home and at primary health care (PHC) facilities were 1.62 times and 2.55 more likely of not practicing exclusive breastfeeding compared to those who give birth at hospitals.

Variables with p-value < 0.25 in bivariate analysis and variables that were substantially important were included in multivariate analysis with backward stepwise selection. The result of multivariate analysis as cited in Table 5, indicates that the mother’s knowledge, self-efficacy, early initiation of breastfeeding and history of COVID-19 infection were associated with exclusive breastfeeding practice after controlling the mother’s education level, employment status, family income, attitude, and perform breast care for milk production. The dominant variable influencing exclusive breastfeeding practice is early initiation of breastfeeding with Odds 78, meaning that the mothers that practice early initiation of breastfeeding were 78 more likely to practice exclusive breastfeeding.

**Table 5:** Logistic regression model of Exclusive breastfeeding practice during pandemic Covid 19 in Depok City.

Variable	Multivariable Analysis		
	AOR	95% CI	P-Value
Education level	4.28	0.62–29.76	0.14
Employment status	0.38	0.08–1.78	0.22
Household income	1.42	0.40–4.98	0.59
Knowledge	77.30	10.18–586.95	0.00
Attitude	0.18	0.03–1.22	0.08
Self-efficacy	5.07	0.98–26.21	0.05
Practice early initiative of breastfeeding	78.11	14.58–418.30	0.00
Ever infected by COVID-19	10.80	1.05–110.90	0.05
Perform breast care for breast milk production	0.52	0.10–2.66	0.43

## Discussion

The prevalence of exclusive breastfeeding was 58.3%, which are slightly higher from the results of the previous research in 2021 in one area in Depok city (56.4%) [19] and the 2017 Indonesia Health Demographic Survey (IHDS) report (52.3%, CI: 0.498–0.548) [10]. However, this figure is still below the figure reported by 2018 Indonesia Basic Health Survey (64.5%) [3]. These differences may be due to the differences in sampling techniques and research locations which only focus on sub-urban areas. This study used cluster sampling technique while Indonesia Basic Health Survey used linear systematic sampling with two stages sampling. Indonesia Basic Health Survey data is also a combination of urban and rural areas.

### Early initiation of breastfeeding

This study found that the dominant factor determining exclusive breastfeeding during COVID-19 pandemic is the practice of early initiation of breastfeeding (EIB). Early initiation of breastfeeding is one of the ways to increase the success of exclusive breastfeeding promoted by WHO and UNICEF. Early initiation of breastfeeding will increase the success of breastfeeding during the first 6 months because early contact between mother and baby will increase the length of breastfeeding twice compared to slow contact. In addition, babies who do not receive EIB can increase their risk of death due to not receiving colostrum so that the baby becomes vulnerable to disease [20]. For instance, according to studies conducted in Jatinangor, Indonesia, exclusive breastfeeding reduces the risk of diarrhea; exclusive breastfeeding has a preventive impact against diarrhea in babies [21].

Research in Ethiopia found that giving colostrum had associated with exclusive breastfeeding, the odds were 2.3 (CI 95%: 1.3–3.9) [22]. It is known that the Infant Mortality Rate (IMR) in Indonesia is still relatively high and infection is still the highest cause, indicating the lack of acquired first immune system from breast milk through the EIB process.

### Mother’s knowledge

The mother’s knowledge level factor was also found to be significantly related to exclusive breastfeeding in the logistic regression results. This finding is in line with the study conducted in Cilangkap, Depok City, which states the knowledge factor as the dominant factor associated with exclusive breastfeeding practices [23]. Appropriate and good knowledge related to exclusive breastfeeding will influence a mother’s attitude and actions to exclusively breastfeed her baby. Besides knowledge, there are others factors related to exclusive breastfeeding as found in one study in Bali, consisted of education, knowledge, perception, husband’s support and exposure to information [24]. The level of maternal knowledge is very influential on exclusive breastfeeding because there is an increase in exclusive breastfeeding if accompanied by an increase in knowledge about exclusive breastfeeding [24]. Mother with a second or higher level of education were 1.2 more likely to perform exclusive breastfeeding than mother who are illiterate or had not attended school [22]. Unfortunately, this study found that half of the respondents had a low level of knowledge about exclusive breastfeeding and early initiation of breastfeeding. This needs to be a concern of health program managers by increasing the promotion of education related to breastfeeding and EIB. Knowledge about breastfeeding is very important because it will be one of the keys to successful implementation of exclusive breastfeeding, especially during the COVID-19 pandemic, mothers worried about the health of their babies, so they need information and supports [25].

### Self-efficacy

The third factor that has a significant relationship with exclusive breastfeeding is self-efficacy towards exclusive breastfeeding. The importance of self-efficacy in breastfeeding mothers to provide exclusive breastfeeding was also found in Zakiah’s study which stated that there was a positive correlation between the self-efficacy of breastfeeding mothers with the length of breastfeeding and exclusive breastfeeding [26]. Another study that found relationship between self-efficacy and exclusive breastfeeding practices was conducted in Ambon. Efforts to increase mothers’ knowledge and awareness about the importance of exclusive breastfeeding practices and the assistance of health workers have the potential to increase mothers’ self-efficacy [27]. The more complete the information obtained by the mother, it will help the mother increase her self-efficacy [28]. During antenatal care, free parental classes for pregnant women, spouses, and family members played a critical role in boosting self-efficacy, confidence and intention for EBF [28]. According to informants in one study in Surabaya, there are mothers who have fears if their breast milk is not enough for the baby, there are also mothers who are afraid that their body shape will change if they provide exclusive breastfeeding [29]. Sources of self-efficacy in the form of verbal persuasion such as encouragement from others (friends, family and breastfeeding counselors) and physiological conditions such as fatigue, stress and anxiety so that the mother’s strong belief in breastfeeding is a determining factor in the success of exclusive breastfeeding [30]. Family members’ most common EBF support was providing mental support or caring for the infant while the mother rested or attended to another responsibility. Mothers who received negative feedback from family members, such as concerns about why breast-fed kids gained less weight, lost confidence and stopped EBF earlier than intended [28].

### Mother’s education

Lawrence Green’s theory suggests that predisposing factors are factors that make it easier for someone to perform health behavior. Age, education, employment status, family income, attitudes, self-efficacy, nutritional status, FFQ scores, and EIB are factors that can encourage behavior. The relationship between one factor and another makes the strength of the theory of health behavior sustainable [30]. For instance, communities, and support from trained counselors and peers, including other mothers and family members, plays a key role of successes exclusive breastfeeding [22].

This result also in line with one research that shows a relationship between maternal education and exclusive breastfeeding practices. The higher the mother’s education level, the chance of exclusive breastfeeding also increases [24]. It is also supported by a research that found the higher a mother’s formal education degree, the greater the value of the possibility for exclusive breastfeeding [9]. Mothers who have good education are easier to absorb diverse information and influence aspects of their thoughts, attitudes, and feelings [24]. Based on Koentjoroningrat’s theory in Anindia et al. (2021), the higher the education, the more knowledge you have. Breastfeeding benefits should be communicated to low-educated mothers, and future breastfeeding programs should focus on maternal and paternal education status [13].

The research in Serang, Banten, Indonesia also showed similar results, with most breastfeeding mothers having low education (43%) and unemployed (62.9%). Furthermore, study with 2017 Nutrition Status Monitoring Survey discovered that unemployed mothers had better EBF practice. This finding is consistent with earlier studies that found employed mothers were less likely to perform EBF [9]. Working mothers tend to have less time to be with their children than non-working mothers, so working mothers will give formula milk as a substitute for breast milk, thus breast milk production will decrease [31].

### Infected with COVID-19

Another factor that included in the model is the experience of infected with COVID-19. This study found some mothers have less knowledge about breastfeeding their babies during the COVID-19 pandemic, as a result, mothers were anxious. Anxiety could also reduce breastmilk production, thus there is an urgent action needed to overcome anxiety. Budiono in Suryaman et al. (2021) stated that people with anxiety will seek efforts to overcome it with various coping mechanisms [32]. As a result, encouraging moms to build nursing intentions and self-efficacy, as well as providing them with important technical skills such as latching and baby placement, is critical for successful EBF [28]. There are some limitations of this study. The cross-sectional nature of the study could not allow us to explore causation beyond associations. There also might be recall bias among respondents who were reporting retrospectively.

## Conclusion and recommendation

The study highlights the importance of improving exclusive breastfeeding practice through early initiation of breastfeeding, mother’s knowledge, education and self-efficacy. Therefore, health promotion and education should emphasize the importance of those factors, supported by the health policy and massive campaign as a key success in exclusive breastfeeding.

## Supporting information

S1 Dataset SPSS data files. (XLSX)

S1 File (PDF)
