# Supplementary material for: Exclusive breastfeeding practice during COVID-19 pandemic in West Java Indonesia: A cross-sectional study
Source: PLoS One. 2024 May 23;19(5):e0303386. doi: 10.1371/journal.pone.0303386 (PMC11115227; doi:10.1371/journal.pone.0303386)
Supplement: S1 File — (PDF) [file pone.0303386.s002.pdf]

UNIVERSITAS PEMBANGUNAN NASIONAL "VETERAN" JAKARTA
KOMISI ETIK PENELITIAN KESEHATAN
Jl. RS. Fatmawati Pondok Labu - Jakarta Selatan 12450
Telp/Fax. 7656971 Ext.123
Homepage: http: www. upnvj.ac.id E-mail : komisietikupnvj@gmail.com

PERSETUJUAN ETIK
ETHICAL APPROVAL
Nomor : 378/VIII/2022/KEPK

Komite Etik Penelitian Kesehatan UPNVJ, dalam upaya melindungi hak asasi dan kesejahteraan subjek penelitian kesehatan dan menjamin bahwa penelitian yang menggunakan formulir survey/registrasi/surveilens/Epidemiologi/Humaniora/Sosial Budaya/Bahan Biologi Tersimpan /Sel punca dan non klinis lainnya berjalan dengan memperhatikan implikasi etik, hukum, sosial dan non klinis lainnya yang berlaku, telah mengkaji dengan teliti proposal penelitian berjudul:
Formulasi Foodbaru dengan Fortifikan Katuk dan Torbangun untuk Peningkatan Produksi ASI dan Status Gizi Bayi

Health Research Ethics Committee UPNVJ, in order to protect the rights and welfare of the health research subjects, and guaranty that the research using survey questionnare/ registry/ surveillance/ epidemiology/ Humaniora/ Social According to ethical, legal, /Biological Materials Stored/stemcells and another non-clinical walk with attention to the social implications, has been thoroughly reviewed the proposal entitled :

Nama Peneliti Utama : **Dr.Apriningsih,SKM.,MKM**
Principal Investigator
Pembimbing / Peneliti Lain :
Supervisor / Other Researcher

Nama Institusi : **Fakultas Ilmu Kesehatan UPN VJ**
Institution
Protokol tersebut dapat disetujui pelaksanaannya.
Hereby declare that the protocol is approved.

Ditetapkan di :Jakarta
Issued in
Tanggal :24 Agustus 2022
Date
Ketua :
Chairman

Prof. Dr. M. Guritno Suryokusumo, dr, SMHS, DEA
NIK: 45113110781

Keterangan/Notes :
Persetujuan etik ini berlaku selama satu tahun sejak tanggal ditetapkan.
Sesuai dengan peraturan yang berlaku di Indonesia, peneliti wajib menyerahkan laporan kemajuan, laporan Kejadian Tidak Diinginkan Serius/KTDS (bila ada), dan laporan akhir pada saat selesai penelitian ke KEPK UPNVJ.
Jika ada perubahan protokol/amanden dan/atau perpanjangan penelitian, harus mengajukan kembali permohonan kajian etik penelitian.
This Ethical clearance is effective for one year from the date specified.
In accordance to Indonesian national regulation, progress, Serious Adverse Events/SAE (if occurred) and final/summary report should be submitted to the EC of UPNVJ.
If there be any modification/amandments and/or extension of the study, the Principal Investigator is required to resubmit the protocol for approval.
